# Mitochondrial dysfunction reactivates **α**-fetoprotein expression that drives copper-dependent immunosuppression in mitochondrial disease models

**DOI:** 10.1172/JCI154684

**Published:** 2023-01-03

**Authors:** Kimberly A. Jett, Zakery N. Baker, Amzad Hossain, Aren Boulet, Paul A. Cobine, Sagnika Ghosh, Philip Ng, Orhan Yilmaz, Kris Barreto, John DeCoteau, Karen Mochoruk, George N. Ioannou, Christopher Savard, Sai Yuan, Osama H.M.H. Abdalla, Christopher Lowden, Byung-Eun Kim, Hai-Ying Mary Cheng, Brendan J. Battersby, Vishal M. Gohil, Scot C. Leary

**Affiliations:** 1 Department of Biochemistry, Microbiology and Immunology, University of Saskatchewan, Saskatoon, Canada.; 2 Department of Biological Sciences, Auburn University, Auburn, Alabama, USA.; 3 Department of Biochemistry and Biophysics, Texas A&M University, College Station, Texas, USA.; 4 Molecular and Human Genetics, Baylor College of Medicine, Houston, Texas, USA.; 5 Department of Laboratory and Pathology Medicine, University of Saskatchewan, Saskatoon, Canada.; 6 Division of Gastroenterology,; 7 Research and Development, Veterans Affairs Puget Sound Health Care System and the; 8 Division of Gastroenterology, Department of Medicine, University of Washington, Seattle, Washington, USA.; 9 Department of Animal and Avian Sciences, University of Maryland, College Park, Maryland, USA.; 10 Department of Biology, University of Toronto Mississauga, Mississauga, Ontario, Canada.; 11 Department of Cell and Systems Biology, University of Toronto, Toronto, Canada.; 12 Institute of Biotechnology, University of Helsinki, Helsinki, Finland.

**Keywords:** Metabolism, Mitochondria

## Abstract

Signaling circuits crucial to systemic physiology are widespread, yet uncovering their molecular underpinnings remains a barrier to understanding the etiology of many metabolic disorders. Here, we identified a copper-linked signaling circuit activated by disruption of mitochondrial function in the murine liver or heart that resulted in atrophy of the spleen and thymus and caused a peripheral white blood cell deficiency. We demonstrated that the leukopenia was caused by α-fetoprotein, which required copper and the cell surface receptor CCR5 to promote white blood cell death. We further showed that α-fetoprotein expression was upregulated in several cell types upon inhibition of oxidative phosphorylation. Collectively, our data argue that α-fetoprotein may be secreted by bioenergetically stressed tissue to suppress the immune system, an effect that may explain the recurrent or chronic infections that are observed in a subset of mitochondrial diseases or in other disorders with secondary mitochondrial dysfunction.

## Graphical abstract

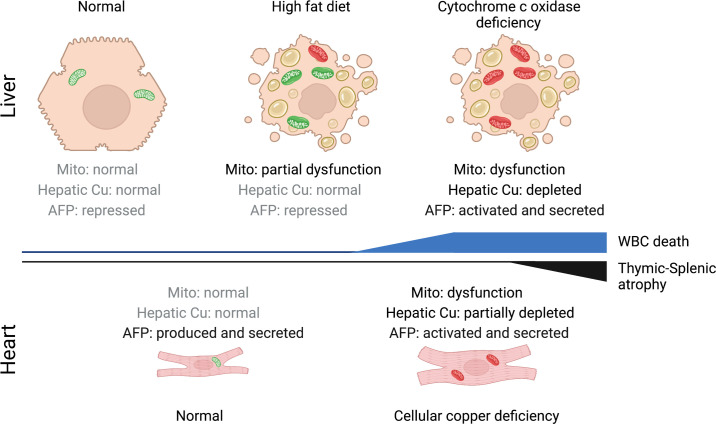

## Introduction

To maintain homeostasis, tissue and organ systems must adapt in unison to varying metabolic challenges (1). These responses involve a cell autonomous component that acts locally at the affected tissue and a complementary, non–cell autonomous component in which secreted factors communicate with other tissues to allow for a coherent systemic response (2, 3). Therefore, to gain insights into systemic physiology and disease etiology it is imperative that we understand how organs sense their functional state and communicate it to distal tissues (4, 5).

A defining feature of human mitochondrial disorders is their tremendous clinical heterogeneity with variable tissue specificity, onset, and severity (6). While the importance of oxidative phosphorylation (OXPHOS) to cell fitness is widely recognized, the cell- and tissue-specific consequences of the mitochondrial dysregulation that leads to the activation of cellular stress responses that ultimately contribute to disease pathogenesis remain poorly characterized. Therefore, a thorough understanding of these molecular mechanisms and how they contribute to pathogenesis is required. Over the last decade, a compendium of studies in human patients and model organisms points to mitochondrial dysfunction as a potent inducer of the integrated stress response (ISR) (7). The ISR is an evolutionarily conserved, intracellular signaling cascade that finely tunes cytoplasmic protein synthesis and nuclear gene expression to preserve homeostasis in response to cellular stress (7). Activating transcription factor 4 (ATF4) integrates the 4 branches of the ISR, directing the expression and secretion of a number of factors. Two of these branches require the translation initiation factors GCN2 and HRI, and each has been shown to communicate mitochondrial dysfunction (8–10). In the context of OXPHOS defects, the enhanced secretion of GDF15 and FGF21 remodels metabolism and both growth factors are recognized as robust biomarkers for mitochondrial disorders (11). However, whether the ISR plays a general role in modulating clinically relevant phenotypes in the molecular pathogenesis of mitochondrial disease remains an outstanding question in the field.

The interplay between mitochondrial dysfunction and the immune system in disease pathogenesis is also poorly understood (12–14), yet patients can present with neutropenia or pancytopenia (15, 16). A comprehensive study investigating immune function and risk of infection further showed that patients with mitochondrial disease had a higher risk of suffering from recurrent infections (17). Here, we establish that an isolated deficiency in cytochrome *c* oxidase (COX) in the liver or heart triggers the secretion of α-fetoprotein (AFP), which then acts systemically to suppress the peripheral immune system by inducing the death of white blood cells (WBCs). AFP therefore appears to play a hitherto underappreciated role in immunomodulatory responses to bioenergetic stress that impact systemic physiology and may contribute significantly to disease progression in patients with mitochondrial disorders who suffer from recurrent infections.

## Results

### Hepatocyte-specific deletion of Sco1 triggers a progressive leukopenia and atrophy of the thymus and spleen.

**Figure 1:**
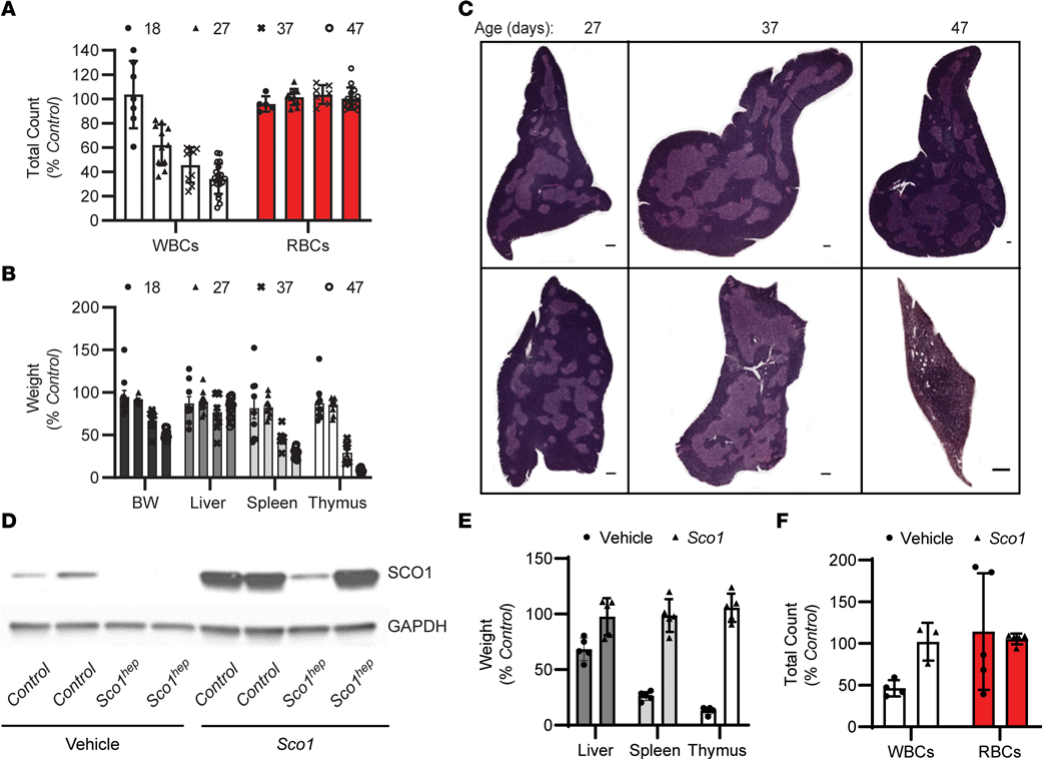
Ablation of *Sco1* expression in hepatocytes results in an unexpected reduction in circulating white blood cell counts and atrophy of the thymus and spleen. (**A**) Progressive leukopenia in *Sco1^hep^* mice (2-way ANOVA with Bonferroni’s post hoc test, *n* = 5–20; *P* < 0.01) and (**B**) disproportionate reduction in the wet weights of the *Sco1^hep^* spleen and thymus at P37 (*n* = 8; *P* < 0.01) and P47 (*n* = 13; *P* < 0.001); 2-tailed Student’s *t* test. (**C**) Selective thinning of the *Sco1^hep^* thymic cortex. Top row: Control. Bottom row: *Sco1^hep^*. Scale bars: 2 mm and 800 μm (P47 *Sco1^hep^* thymus). (**D**) Adenoviral restoration of SCO1 expression in the liver leads to (**E**) rescue of splenic and thymic atrophy (*n* = 5) and (**F**) normalization of WBC counts (*n* = 3–5). Mice were injected on P21 via cardiac puncture with vehicle or helper-dependent adenovirus harboring a *Sco1* cDNA under the control of a liver-specific promoter and harvested at P47. Control, wild-type littermates; BW, body weight; WBC and RBC, white and red blood cells, respectively.

We previously demonstrated that mice lacking the copper chaperone *Sco1* in hepatocytes (*Sco1^hep^*) develop normally during the first month of life, but subsequently fail to thrive and have a median life expectancy of 70 days (18). Surprisingly, adult *Sco1^hep^* mice exhibit a severe leukopenia and profound atrophy of the spleen (18); however, it is unclear whether these phenotypes are a direct consequence of ablating *Sco1* expression in hepatocytes or a secondary consequence of liver failure. Therefore, to understand whether hepatic deletion of *Sco1* triggers systemic signaling that affects cells and organs of the immune system, we measured complete blood cell (CBC) counts, quantified organ mass, and examined the ultrastructure of the thymus in *Sco1^hep^* mice and age-matched littermate controls on postnatal day 18 (P18), P27, P37, and P47 (Figure 1). This sampling schedule was selected because at P27 *Sco1^hep^* mice are outwardly indistinguishable from Control littermates, yet their livers already exhibit a severe, combined COX and copper deficiency (18). We found that the WBC count deficiency in terminal blood drawn from *Sco1^hep^* mice was already manifest at P27 and progressively worsened thereafter (Figure 1A). Notably, we did not detect any changes in red blood cell (RBC) counts (Figure 1A). The onset of the leukopenia preceded any loss in body weight and was ultimately accompanied by the disproportionate atrophy of the spleen and thymus relative to other organs (Figure 1B). These data suggest that the effects on the peripheral immune system in *Sco1^hep^* mice are not secondary to liver failure, and clearly establish that the leukopenia is manifest prior to the atrophy of the thymus and spleen.

To gain further insight into the underlying atrophy of the thymus, we examined thymic ultrastructure using standard hematoxylin and eosin (H&E) staining (Figure 1C). While the thymi of Control and *Sco1^hep^* mice were similar at P27, histological analysis of the P37 *Sco1^hep^* thymus revealed a selective thinning of the cortex (Figure 1C) and the presence of tingible body macrophages (Supplemental Figure 1A; supplemental material available online with this article; https://doi.org/10.1172/JCI154684DS1). Additional atrophy at P47 was accompanied by a further reduction in the ratio of cortex to medulla, disruption of the cortical-medullary boundary, and increased vascularity (Figure 1C and Supplemental Figure 1A). These data together with our previous findings (18) suggest that loss of *Sco1* in hepatocytes has a profound effect on the peripheral immune system, and that atrophy of the thymus and spleen in *Sco1^hep^* mice ultimately contributes to the progressive severity of the leukopenia.

### The liver secretes an immunosuppressive factor in response to bioenergetic stress caused by mitochondrial dysfunction.

While characterization of the albumin Cre recombinase driver we used for *Sco1* deletion has demonstrated that loxP-mediated gene excision is largely restricted to hepatocytes (19), we wanted to ensure that the observed immune phenotypes are indeed directly attributable to the loss of SCO1 function in the liver. We therefore sought to restore *Sco1* expression in the liver of *Sco1^hep^* mice using a helper-dependent adenoviral approach (20, 21). After establishing that intracardiac administration was superior to intraperitoneal (i.p.) injection for specific delivery of helper-dependent adenovirus (HdAD) to the liver relative to other peripheral organs (Supplemental Figure 1B), we injected 21- to 24-day-old *Sco1^hep^* mice and Control littermates with vehicle or an HdAD containing a *Sco1* cDNA under the control of a liver-specific *PEPCK* promoter. SCO1 abundance was significantly increased in the livers of both Control and *Sco1^hep^* mice (Figure 1D), which rescued the copper deficiency and the levels of the cellular copper importer CTR1 (Supplemental Figure 1, C and D). Zinc and iron levels were also normalized in the livers of *Sco1^hep^* mice injected with adenovirus (Supplemental Figure 1C). Critically, adenovirus administration normalized body and organ weights and restored WBC counts in *Sco1^hep^* mice (Figure 1, E and F). These data show that the immunosuppressive effects of *Sco1* loss of function are specific to its role in hepatocytes and not attributable to aberrant Cre recombinase expression in other cell types.

**Figure 2:**
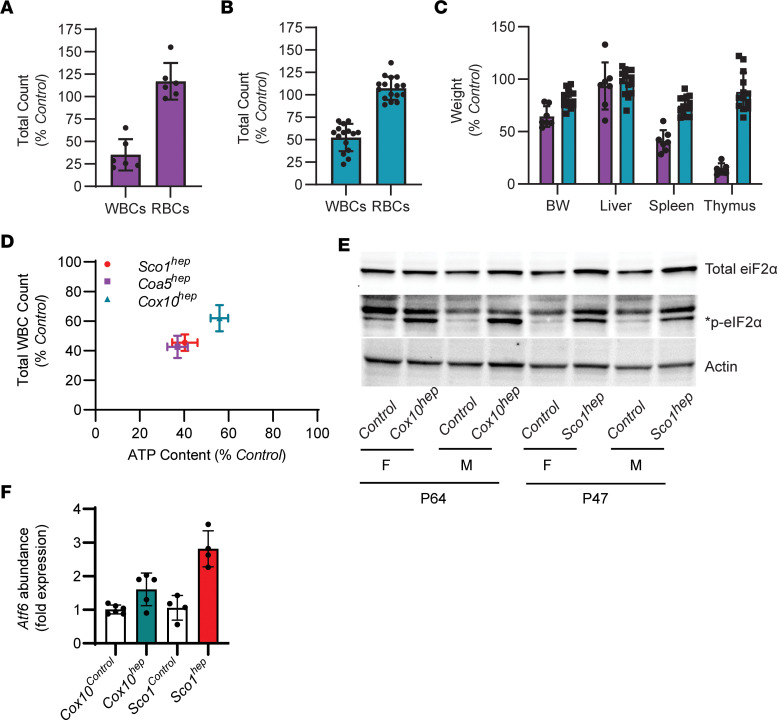
The reduction in peripheral WBC counts in hepatocytes is positively correlated with the bioenergetic deficit in the liver. (**A**) *Coa5^hep^* (*n* = 6; *P* < 0.001) and (**B**) *Cox10^hep^* (*n* = 15–16; *P* < 0.01) mice also exhibit a significant leukopenia. (**C**) *Coa5^hep^* mice (purple bars) have a disproportionate reduction in the wet weight of the spleen (*n* = 6; *P* < 0.01) and thymus (*n* = 5; *P* < 0.001), while *Cox10^hep^* mice (blue bars) exhibit significant yet milder atrophy of the spleen (*n* = 14; *P* < 0.05). Significance was assessed using a 2-tailed Student’s *t* test (**A**–**C**). (**D**) Total WBC counts are positively correlated with liver ATP content in all 3 *hep* mouse models (linear regression *R*^2^ = 0.99; *P* = 0.001). P47 *Sco1^hep^* and P64 *Cox10^hep^* livers have higher levels of (**E**) the phosphorylated form of eIF2α, an ISR marker, and (**F**) the *Atf6* transcript, a marker of ER stress (1-way ANOVA with Tukey’s post hoc test, *n* = 4–6; *P* < 0.005, *Sco1^hep^* model; *P* < 0.02, *Cox10^hep^* model).

To determine whether the immunosuppressive effect we observe is unique to the *Sco1^hep^* mouse model, we generated 2 additional hepatocyte-specific knockout lines that lacked the COX assembly factor cytochrome *c* oxidase assembly factor 5 (*Coa5*) or *Cox10*. Unlike *Sco1^hep^* mice, and in agreement with existing literature, hepatocyte-specific deletion of *Coa5* or *Cox10* (22) was not lethal (Supplemental Figure 2, A and B). Therefore, *Coa5^hep^* and *Cox10^hep^* mice were sampled on P77 and P64, respectively, as they display the greatest body weight difference at these time points when compared with Control littermates (Supplemental Figure 2, A and B). Both *Coa5^hep^* (Supplemental Figure 2C) and *Cox10^hep^* (18) mice exhibited a severe, combined COX and copper deficiency in the liver, and a significant leukopenia in the absence of changes in RBC counts (Figure 2, A and B). Copper levels in the circulation were higher in both *hep* models (Supplemental Figure 2D), while atrophy of the thymus and spleen was marked in *Coa5^hep^* mice and relatively modest in *Cox10^hep^* animals (Figure 2C).

To evaluate whether the varying severity of the leukopenia we observe across our mouse models is attributable to differences in hepatic bioenergetic status, we next quantified total ATP levels in age-matched Control and *hep* livers and plotted them against total WBC counts. These analyses revealed a significant, positive correlation between hepatic ATP content and WBC counts (Figure 2D). Consistent with the idea that perturbed energy homeostasis is associated with mitochondrial dysfunction, *Sco1^hep^* and *Cox10^hep^* livers exhibited a significant increase in the abundance of the phosphorylated form of eIF2α, a key player in the ISR (23, 24) (Figure 2E). Given that the ISR is a robust intracellular signaling cascade that can integrate both endoplasmic reticulum (ER) and mitochondrial dysfunction (25), we also quantified the transcript abundance of ATF6, a well-known transcription factor that is activated upon ER stress, and found that it was upregulated in the *hep* livers of both the *Sco1* and *Cox10* mouse models (Figure 2F).

Taken together, our findings from 3 unique mouse models argue that ER stress and mitochondrial dysfunction in the liver triggers the secretion of a factor that suppresses the peripheral immune system. Our data further suggest that the severity of the leukopenia is a direct reflection of the extent to which the *hep* liver is bioenergetically stressed as a result of impaired mitochondrial function.

### Hepatic mitochondrial dysfunction leads to the secretion of the immunosuppressive factor AFP.

**Figure 3:**
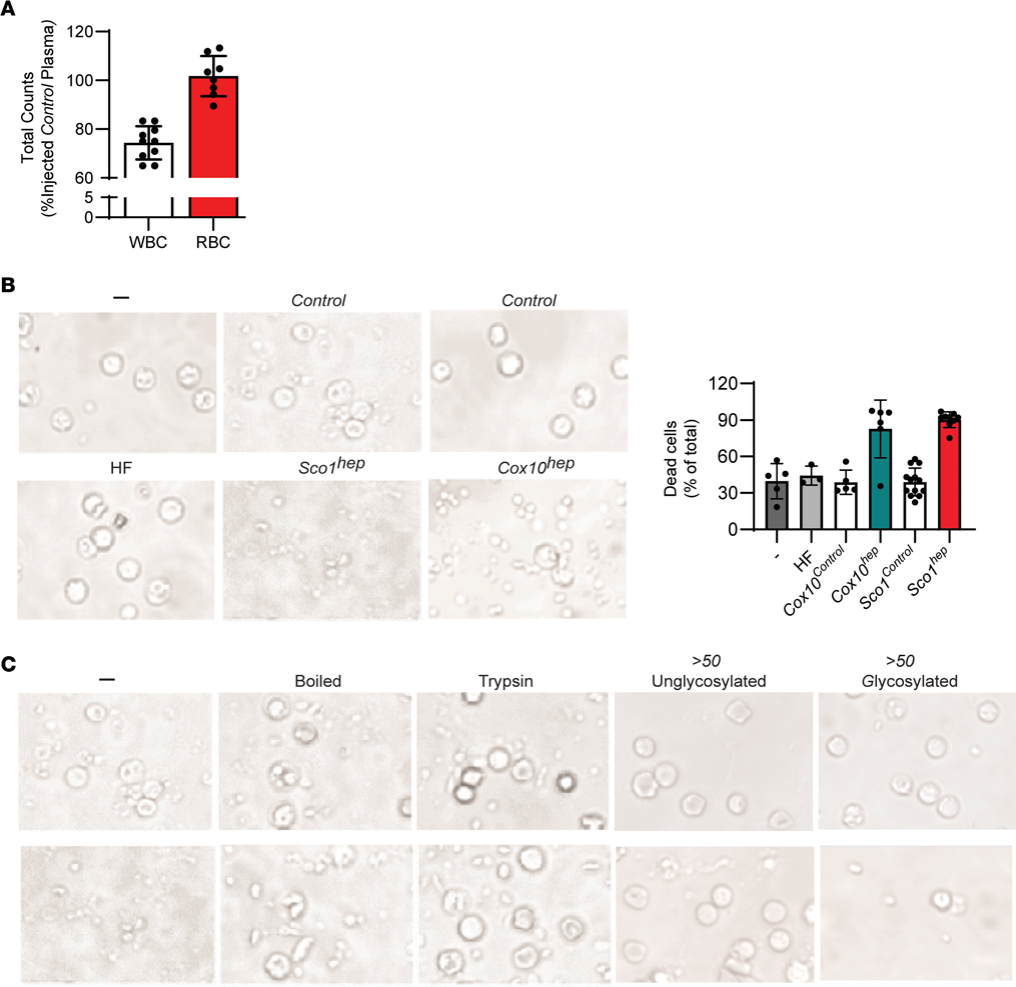
A glycoprotein secreted by mice with altered mitochondrial function in the liver is responsible for the observed reduction in peripheral WBC counts. (**A**) Total WBC counts are reduced in Control mice injected with *Sco1^hep^* plasma relative to those injected with Control plasma (*n* = 10; *P* = 0.02 by 2-tailed Student’s *t* test). (**B**) The viability of PBMCs isolated from Control mice is significantly reduced when they are cultured with *Sco1^hep^* or *Cox10^hep^* plasma. PBMC viability is unaffected by exposure to plasma from mice fed a high-fat (HF) diet. Bar graph on right summarizes the operator-blinded quantitation of dead cells per treatment group expressed as a percentage of the total number of cells (live and dead) per image (*n* = 3–13 per treatment group, *P* < 0.0001, both *hep* models versus PBMCs cultured in FBS alone [–], HF, or Control treatment). (**C**) PBMC viability is rescued if *Sco1^hep^* plasma is boiled or treated with trypsin. Fractionation based on size and the presence of a glycan revealed that the factor(s) that reduces PBMC viability is present in a >50 kDa glycoprotein fraction. Scale bars: 50 μm (**B** and **C**). The Control and *Sco1^hep^* images in panel **B** are identical to those denoted as “–” in **C** because they were part of the same experimental series.

To establish that a secreted factor is responsible for the immune phenotypes we observe as a result of mitochondrial dysfunction in the liver, we serially administered Control or *Sco1^hep^* plasma to 21- to 24-day-old Control mice over 28 days via tail vein injection. Mice injected with *Sco1^hep^* plasma exhibited a significant leukopenia relative to those injected with Control plasma (Figure 3A), independent of alterations in thymic mass (Supplemental Figure 3A), emphasizing that the leukopenia in our *hep* models does not require atrophy of the spleen or thymus. Taken together, these findings are consistent with a model whereby the *Sco1^hep^* liver is secreting a factor into the blood that is suppressing the peripheral immune system.

A number of secreted molecules, including proteins, lipids, nucleic acids, and metabolites, could account for the observed immune phenotypes (26, 27). To distinguish between these possibilities, we performed an in vitro peripheral blood mononuclear cell (PBMC) viability assay with Control and *hep* plasma sources. PBMCs grown in RPMI containing Control plasma are indistinguishable from those grown in media supplemented with fetal bovine serum (FBS) (vs. Control; Figure 3B and Supplemental Figure 3D). In contrast, the viability of PBMCs cultured with *Sco1^hep^* or *Cox10^hep^* plasma was significantly diminished (Figure 3B). Because *Sco1^hep^* livers have profound alterations in lipid metabolism reminiscent of nonalcoholic fatty liver disease (NAFLD) (18), we next addressed the possibility that the observed effects in PBMC cultures were attributable to one or more lipid species. We found that PBMC viability was unaffected by culturing with plasma isolated from mice fed a high-fat (HF) diet (Figure 3B and Supplemental Figure 3D) (28) that had fatty livers without any discernible changes in their hepatic metal ion content or the abundance of core subunits of each OXPHOS enzyme complex (Supplemental Figure 3, B and C). In contrast, boiling or trypsin treatment of *Sco1^hep^* plasma abolished its negative effect on PBMC viability (Figure 3C), suggesting that the secreted factor is a protein. We therefore fractionated plasma proteins based on size and the presence of a glycan, and found that the bioactivity was retained in a >50 kDa glycoprotein–containing fraction (Figure 3C).

**Figure 4:**
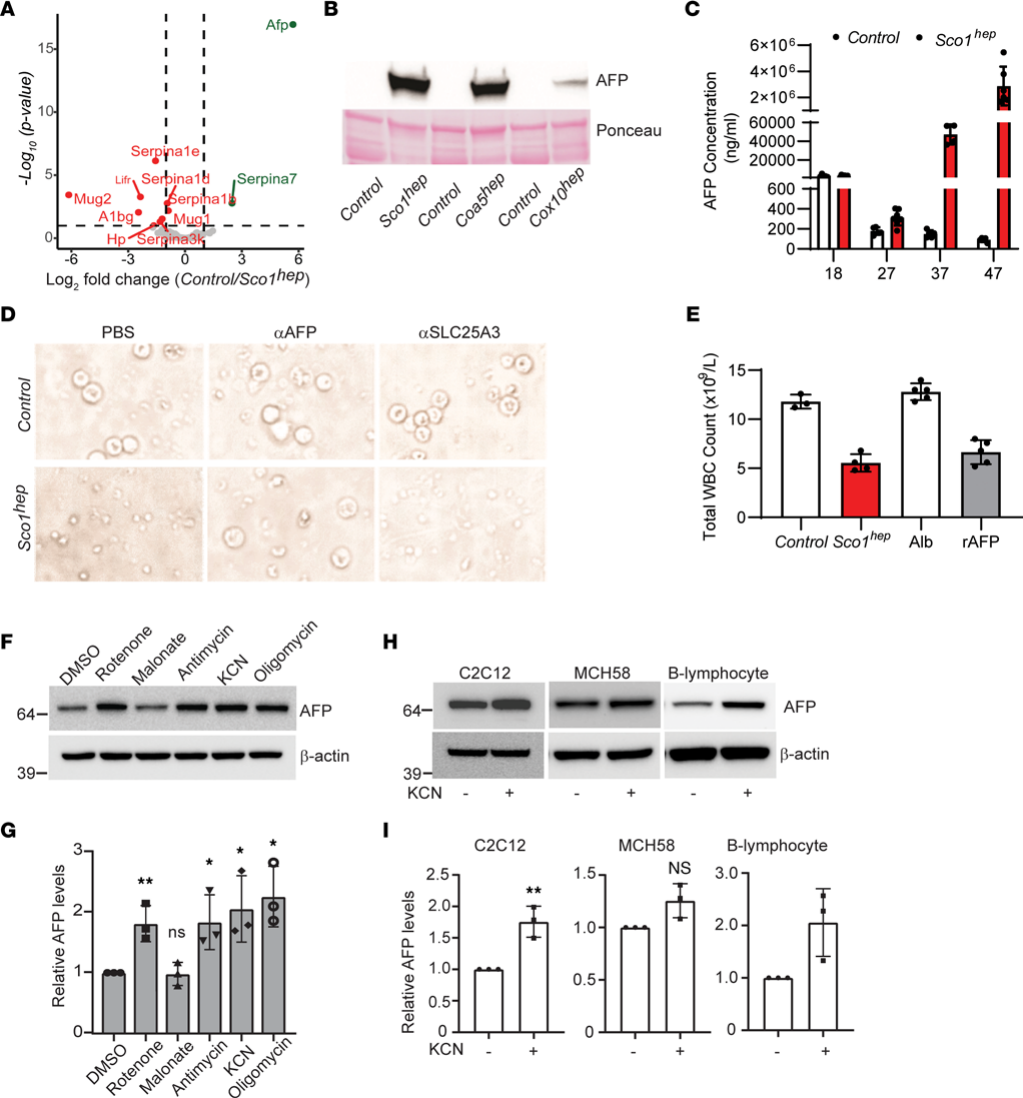
AFP expression is increased in response to impaired organelle function and is directly responsible for the leukopenia observed in several mitochondrial disease models. (**A**) AFP is significantly enriched in *hep* relative to Control plasma. Volcano plot with dotted lines indicating a 2-fold change and adjusted *P*-value significance threshold of 0.1. A green or red symbol indicates a protein whose abundance is significantly up- or downregulated, respectively. Gray symbols denote proteins whose abundance is not significantly different (NS) between the 2 groups. (**B**) AFP abundance is markedly upregulated in *Sco1^hep^*, *Coa5^hep^*, and *Cox10^hep^* plasma. Plasma was pooled (minimum of 2 males and 2 females per pool) and depleted of immunoglobulins and albumin prior to Western blotting. The Ponceau S–stained membrane indicates relative loading across lanes. (**C**) AFP progressively accumulates in *Sco1^hep^* plasma (*n* = 4–7; P27, *P* < 0.05; P37, *P* < 0.0001; P47, *P* < 0.0001). (**D**) PBMC viability is rescued by immunodepleting AFP from *Sco1^hep^* plasma. Culture media supplemented with PBS and plasma incubated with αSLC25A3, an antibody isotype control, served as internal controls. Original magnification, ×60 (same scale as in Figure 3, B and C; Figure 5, D and E; and Figure 6, C and D). (**E**) Control mice develop a leukopenia following serial injection with *Sco1^hep^* plasma (*n* = 3–4; *P* < 0.01) or 1 μg of recombinant AFP (rAFP) (*n* = 5; *P* < 0.01). Control mice injected with Control plasma or albumin served as internal controls. (**F** and **G**) Inhibition of the mitochondrial respiratory chain elevates AFP abundance in C2C12 myoblasts. (**H** and **I**) AFP levels increase in C2C12 myoblasts and human B lymphoblasts upon inhibition of COX. For panels **F**–**I**, β-actin was used as a loading control and data are shown as mean ± SD (*n* = 3). *P < 0.05, **P < 0.005. Significance was assessed using a 2-tailed Student’s *t* test (**C**, **E**, **G**, and **I**). NS, not significant.

To identify the secreted factor, the >50 kDa glycoprotein–containing Control, *hep*, and HF plasma fractions were analyzed by quantitative mass spectrometry (MS) (Figure 4A). We reasoned that the immunosuppressive factor would be absent in HF plasma, enriched in *hep* plasma, and more abundant in *Sco1^hep^* than *Cox10^hep^* plasma, given the relative severity of the leukopenia in each model. Of the 2 upregulated hits, only AFP met those criteria (Figure 4A and Supplemental Figure 4A). Consistent with our MS results, AFP abundance was elevated in P47 *Sco1^hep^*, P77 *Coa5^hep^*, and P64 *Cox10^hep^* plasma (Figure 4B), and its circulating levels were significantly higher in *Sco1^hep^* than Control plasma at P27 and continued to increase as the severity of the leukopenia worsened over time (Figure 4C). In fact, the roughly 2,000-fold difference in AFP abundance between Control and *Sco1^hep^* plasma in P47 mice was mirrored at the transcript level in the *Sco1^hep^* liver (Supplemental Figure 4B), emphasizing that the liver is the principal organ responsible for secreting AFP into the circulation.

To further validate AFP as the active component of our signaling axis, we investigated the effect of manipulating its abundance on WBC viability both in vitro and in vivo. The viability of PBMCs cultured in *Sco1^hep^* plasma was rescued upon immunodepletion of AFP but not by pretreatment of plasma with anti-SLC25A3, an isotype antibody control (Figure 4D). Supplementation of standard media with recombinant AFP (rAFP) alone also induced PBMC death (Supplemental Figure 4C). Corroborating our in vitro findings, serial injection of 21- to 24-day-old Control mice with rAFP, but not with the closely related family member albumin, resulted in a leukopenia of comparable severity to that seen in Control mice injected with *Sco1^hep^* plasma (Figure 4E). These data collectively argue that AFP secreted by the liver is directly responsible for the unexpected immunosuppression we observe in our *hep* mouse models of mitochondrial disease.

### AFP can be produced by nonhepatic models of mitochondrial dysfunction and requires copper for its immunosuppressive activity.

AFP is highly expressed during embryonic development and is also secreted by the yolk sac, stomach, and cells of the intestine (29). While its expression is repressed after birth by epigenetic mechanisms (30, 31), it is known that the adult liver reactivates the *AFP* locus and secretes the protein in response to fibrosis, hepatitis, and several hepatic cancers (32–34). We therefore investigated whether mitochondrial dysfunction is a general trigger for AFP overexpression in other cell types. To test this idea, we treated the murine myoblast C2C12 cell line with pharmacological agents to inhibit specific OXPHOS complexes. Indeed, specific inhibition of COX (complex IV) and complex I, III, or V resulted in a modest but significant increase in steady-state AFP levels (Figure 4, F and G). To determine whether this is a cell-type-dependent phenotype, we treated 2 additional cell types with a complex IV inhibitor and found that skeletal myoblasts and B lymphocytes, but not fibroblasts, upregulated AFP production in response to COX inhibition (Figure 4, H and I). These data suggest that in addition to hepatocytes, several other cell types are able to increase AFP production in response to mitochondrial dysfunction. To expand upon our pharmacological analyses, we investigated whether AFP expression is upregulated in the muscle of a whole-animal model with secondary mitochondrial dysfunction. We found that COX-deficient murine hearts lacking the high-affinity copper importer CTR1 (35) indeed have significantly higher levels of *Afp* transcript (Supplemental Figure 5A) and protein (Supplemental Figure 5B), along with elevated levels of the ISR marker phospho-eIF2α (Supplemental Figure 5B) when compared with the hearts of Control littermates. Taken together, these findings indicate that mitochondrial dysfunction triggers reactivation of AFP expression in diverse cell types and tissues.

**Figure 5:**
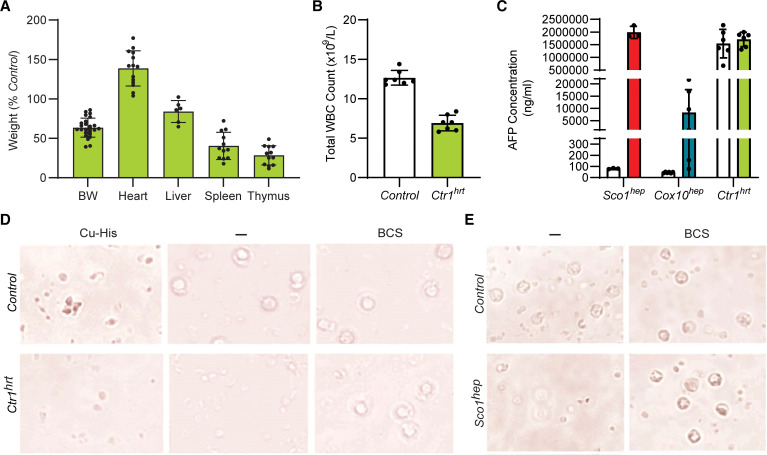
AFP requires copper to promote the death of peripheral WBCs. (**A**) *Ctr1^hrt^* mice exhibit disproportionate atrophy of the spleen (*n* = 12; *P* < 0.01) and thymus (*n* = 12; *P* < 0.01) and have a (**B**) leukopenia (*n* = 7; *P* < 0.01). Significance was assessed using a 2-tailed Student’s *t* test (**A** and **B**). (**C**) Plasma AFP levels are comparable in P10 Control and *Ctr1^hrt^* plasma mice. Relative levels of AFP in *Sco1^hep^*, *Cox10^hep^*, and age-matched littermate Control plasma were quantified at the same time and are shown here for comparative purposes (*n* = 3–6). (**D**) PBMC viability is adversely affected by *Ctr1^hrt^* plasma, an effect that can be rescued by adding the copper chelator bathocuproine sulphonate (BCS) to the media. Viability of PBMCs is also reduced if copper-histidine (Cu-His) is added to media containing P10 Control plasma. (**E**) BCS also negates the negative effect of *Sco1^hep^* plasma on PBMC viability. Scale bars: 50 μm (**D** and **E**).

Several proteoforms of AFP are known to be present in the general circulation and at least some of these have the ability to bind a variety of ligands, including copper (36). Therefore, an intriguing possibility is that the leukopenia in *hep* mice is caused by a specific conformer(s) constituting a fraction of the total plasma AFP pool. To address this possibility, we took further advantage of the *Ctr1^hrt^* mouse model because its COX-deficient heart is known to communicate with the liver, leading to a significant hepatic copper deficiency (35). Like several of our *hep* models, P10 *Ctr1^hrt^* mice exhibited disproportionate atrophy of the spleen and thymus (Figure 5A) and a significant leukopenia (Figure 5B). Due to their young age, circulating AFP levels were comparable in Control and *Ctr1^hrt^* animals (Figure 5C); however, only *Ctr1^hrt^* plasma was capable of killing PBMCs (Figure 5D). This observation is consistent with the idea that AFP abundance alone does not explain its immunosuppressive properties. In fact, *Ctr1^hrt^* and *Sco1^hep^* plasma pretreated with the Cu(I)-specific chelator bathocuproine disulfonic acid (BCS) prevented PBMC death (Figure 5, D and E), whereas the addition of copper salts to the culture media enabled the AFP-rich, age-matched (P10) Control plasma to stimulate PBMC death (Figure 5D). Consistent with these observations, PBMC viability was unaffected upon culturing with recombinant AFP produced and isolated from *E*. *coli* (Supplemental Figure 5C), where free copper is known to be very scarce in the cytosol (37). Collectively, these data argue that AFP requires copper to induce cell death in WBCs and cause a leukopenia.

### AFP promotes the death of mouse and human PBMCs via the receptor CCR5.

**Figure 6:**
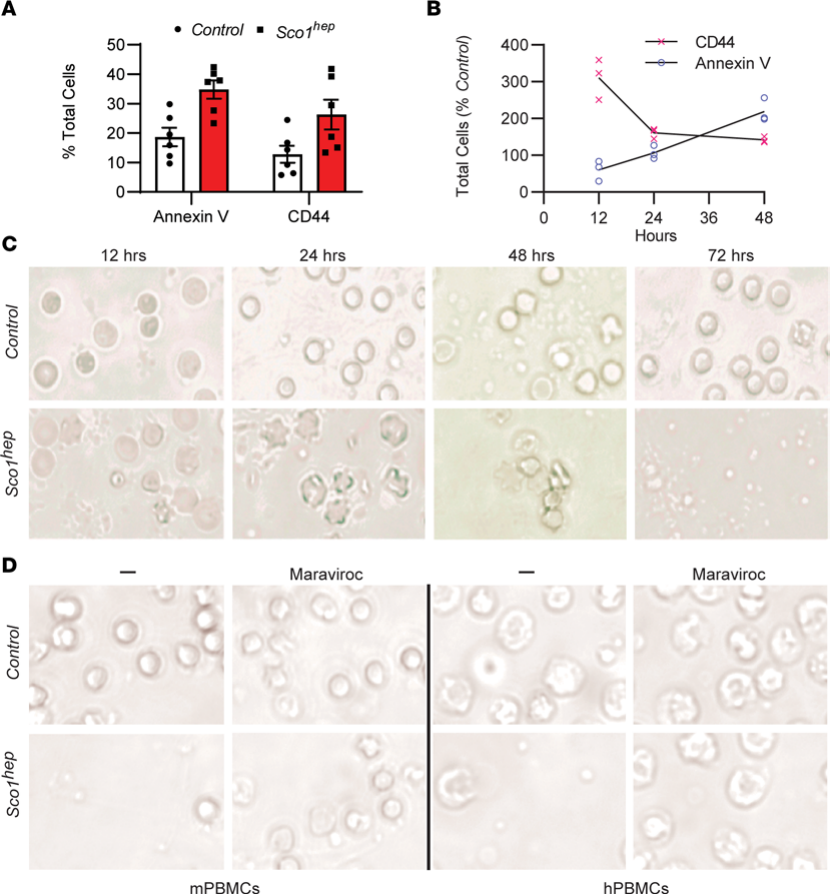
AFP promotes activation and apoptosis of both mouse and human WBCs via the cell surface receptor CCR5. (**A**) PBMCs isolated from peripheral blood of *Sco1^hep^* mice have an increased number of cells that stain positive for the activation marker CD44 and the apoptotic marker annexin V when compared with PBMCs isolated from Controls (*n* = 6). (**B**) PBMCs isolated from Control mice that were cultured in media containing *Sco1^hep^* plasma are activated earlier and demonstrate a progressive increase in cell surface expression of the apoptotic marker annexin V relative to PBMCs cultured in Control plasma (*n* = 3). (**C**) Cells analyzed in **B** show blebbing (a sign of apoptosis) as early as 12–24 hours in culture and loss of cellularity after 72 hours in culture in response to culturing with *Sco1^hep^* but not Control plasma. (**D**) Human PBMC viability is also reduced in media containing *Sco1^hep^* plasma, and can be rescued with the CCR5 antagonist maraviroc. mPBMCs and hPBMCs, PBMCs of mouse and human origin, respectively (mPBMCs, *n* = 3; hPBMCs *n* = 2; each replicate contained a triplicate for each experimental condition). Scale bars: 50 μm (**C** and **D**).

To establish that AFP causes a leukopenia by stimulating WBC death, we isolated PBMCs from P47 Control and *Sco1^hep^* mice and cells positive for CD44, a marker of activation, and annexin V, an indicator of cell death. Both markers were significantly elevated (CD44, *P* = 0.017; annexin V, *P* = 0.003) in *Sco1^hep^* PBMCs when compared with Control PBMCs (Figure 6A and Supplemental Figure 6), consistent with the idea that AFP indeed stimulates WBC death. To confirm our in vivo observations and further delineate the temporal relationship between activation and cell death in the presence of bioactive AFP, we conducted a time-course experiment using naive, wild-type PBMCs cultured in Control or *Sco1^hep^* plasma. At 12 hours, CD44 staining was significantly higher (*P* = 0.02) in PBMCs grown in media containing *Sco1^hep^* plasma, while annexin V staining was similar in Control and *Sco1^hep^* cultures (Figure 6, B and C). Annexin V–positive cell numbers then increased significantly in the *Sco1^hep^* culture over the next 36 hours (48 hours, *P* = 0.03; Figure 6B), indicating that activated PBMCs were indeed dying.

It has been proposed that AFP exerts its immunosuppressive effects by binding to various cation channels and classes of receptors that include mucin, scavenger, lysophospholipid, and chemokine receptors (38–43). To date, however, AFP has only been shown to bind directly to the chemokine receptor CCR5 (44). We therefore cultured PBMCs in Control or *Sco1^hep^* plasma alone or in media that also contained the CCR5 antagonist maraviroc (45) and found that receptor blocking rescued cell viability (Figure 6D). To determine whether AFP from our *hep* mouse models of mitochondrial disease also acts in an immunosuppressive manner on human cells via the same mechanism, we repeated the experiment and found that pharmacologically blocking CCR5 ablated the ability of *Sco1^hep^* plasma to kill human PBMCs (Figure 6D). Taken together, our data argue that AFP requires both copper and CCR5 to activate and kill WBCs to induce a leukopenia. Our findings further establish that this immunosuppressive mechanism is also capable of triggering the death of human PBMCs and suggest that tissue release of a specific AFP conformer in response to mitochondrial dysfunction provides a mechanism to suppress the peripheral immune system.

## Discussion

Here, we uncover a molecular mechanism by which a primary mitochondrial dysfunction in the liver compromises the peripheral immune system, revealing the basis of a surprising inter-tissue signaling circuit. We demonstrate that a defect in OXPHOS in the postnatal mouse liver induces robust secretion of AFP that, in concert with copper, activates cell death in leukocytes by interacting with the cell surface receptor CCR5. In turn, this leads to a progressive leukopenia and atrophy of the thymus and spleen. Patients with inherited mitochondrial disorders are well known to be susceptible to acute febrile infections (46) and in some cases manifest with congenital neutropenia (47); however, a molecular basis for these phenotypes has been lacking until now. Together, our findings identify an immunosuppressive mechanism that could account for why some groups of patients with mitochondrial dysfunction are vulnerable to infections. It may also explain why patients with elevated circulating AFP levels owing to ataxia telangiectasia or other cerebellar ataxias with suspected mitochondrial involvement are also prone to recurrent infection (48, 49). However, considering the tremendous clinical heterogeneity of mitochondrial diseases it is likely that additional mechanisms contribute to immunosuppression in a context-dependent manner that have yet to be identified.

AFP is a major serum protein expressed by the developing embryo that is under tight temporal and spatial regulation (30, 50). After birth, multiple genetic factors coordinate a conserved developmental program to repress transcription via elements upstream of the gene promoter (31, 51, 52). However, liver dysfunction in adults can reactivate AFP expression in hepatocytes, most notably with hepatic cellular carcinoma (HCC) and fibrosis (32, 34). Though the precise functional consequence of AFP secretion in these disease settings is poorly understood, it has been suggested to increase hepatocyte cell proliferation in HCC. In the context of mitochondrial dysfunction, hyperplasia of hepatocytes is not observed (18) nor are high circulating levels of AFP alone toxic to leukocytes. Instead, the leukocyte toxicity requires AFP and copper, a known ligand of this serum protein (36).

Copper is an essential metal that acts as a cofactor for select cellular enzymes catalyzing redox reactions. This trace element is obtained from the diet and then systemically distributed to meet cellular demand. In mammals, the liver acts as the principal storage tissue to coordinate systemic copper levels, a function that is of utmost importance in early postnatal life and in response to copper handling defects in other tissues. Intracellular copper is trafficked by a series of dedicated chaperones that form an effective delivery pathway to target enzymes for metalation or labile pools for storage (53). Many of the central factors for copper transport have been elucidated and disruptions to these steps can exert devastating effects on human health. In our mouse models of mitochondrial dysfunction, we observe a signature of disrupted copper homeostasis in the liver and a responsive cascade of subsequent compensatory events. This response initiates with the progressive loss of the copper transporter CTR1 in the liver via proteasomal degradation, effectively reducing copper uptake (18). However, a shift to enhance copper efflux from the tissue likely follows, given that the levels of copper and the copper-dependent ferroxidase ceruloplasmin are both elevated in *hep* plasma (18). Consistent with an important role for hepatic copper mobilization in the observed immune phenotypes, the liver of *Ctr1^hrt^* mice upregulates ATP7A expression and pumps copper into the circulation as a consequence of loss of CTR1 function in the heart, a response that renders it copper deficient relative to the liver of wild-type littermates (35).

To reconcile our findings, we propose the following model. The induction of AFP synthesis in response to mitochondrial dysfunction coincides with the cellular response to increased copper efflux into the secretory system. Although the exact role of copper in potentiating the immunosuppressive properties of AFP is currently unclear, we speculate that for AFP to be cytotoxic to leukocytes it chelates copper during secretion from hepatocytes. Central to this model is the role of mitochondria in regulating liver copper homeostasis (18, 54–56). We hypothesize that the coupling of these two molecular events is required for the observed leukopenia.

Our model accounts for the apparent inert activity of high circulating levels of AFP in the early postnatal period and the absence of a leukopenia with HCC. Further, the model makes clear predictions that disruptions to copper efflux into the secretory system would ablate the AFP-driven leukocyte toxicity. Copper delivery to the secretory system is mediated via ATOX1 to the ATPase copper pumps, ATP7A and ATP7B (57). Pathogenic variants in *ATP7A* and *ATP7B* manifest as Menkes and Wilson disease, respectively (58, 59). In line with our model, neither disorder is characterized by immune suppression or neutropenia, even though severe liver pathology is a hallmark of Wilson disease (60). Mitochondrial signaling has previously been shown to promote cellular copper efflux (55, 56), and the most severe leukopenias in our *hep* models coincide with higher levels of copper in the circulation. While these findings support the notion that sufficient ATP7A and ATP7B remains localized within the secretory system to account for the observed phenotypes in liver experiencing mitochondrial dysfunction, future investigations will be required to untangle the trafficking dynamics of the efflux transporters in hepatocytes under these conditions.

A key question remains, which is how is the transcriptional suppression of AFP expression released? Although we observe ISR activation upon mitochondrial dysfunction in the liver, it may be that it is not the signaling cascade that regulates AFP induction. ISR activation is emerging as a robust stress response to mitochondrial OXPHOS dysfunction in patients and in animal models of these diseases (10, 11, 61–63). The signaling cascade leads to the induction and secretion of 2 growth factors, GDF15 and FGF21, that remodel cellular metabolism in response to nutritional and metabolic disturbances. Thus, while induction of this stress response is not surprising in our mouse models, ISR activation in other mitochondrial disorders caused by OXPHOS dysfunction is not always accompanied by increased AFP expression or a leukopenia (64, 65). This suggests a cell-type-specific mechanism whereby severe disruptions to hepatocyte homeostasis override the transcriptional repression of AFP. We propose instead that the central role of mitochondrial signaling in regulating copper homeostasis in the liver likely underpins the mechanism for AFP immunosuppression. The basis of this pathway opens up an exciting new avenue for the role of mitochondria in intracellular signaling.

Another outcome of our study is the identification of a potential new biomarker of mitochondrial dysfunction in the liver. Currently, GDF15 and FSF21 are robust biomarkers detected in the circulation of patients with specific classes of mitochondrial disorders, particularly with skeletal muscle involvement (11, 62, 63, 66, 67). Interestingly, these biomarkers were first identified in mouse models of mitochondrial myopathy, including those with a *Cox10* deficiency (68). This highlights the promise of translating discoveries derived from mouse models of mitochondrial disorders into robust clinical practice for noninvasive patient diagnostics. Future studies with large cohorts of patients screened for circulating AFP expression and bioactivity will need to be performed, as previous experience emphasizes that not all mitochondrial diseases induce GDF15 and FGF21 expression (11), further arguing that there is specificity in the systemic response to mitochondrial dysfunction.

In conclusion, we identify what we believe is a novel tissue crosstalk mechanism whereby the homeostatic role of mitochondria in coordinating hepatic copper homeostasis intersects with the function of the peripheral immune system. Diet could also be a key factor in modulating the response, particularly with foods rich in copper. Future studies will explore the role of this leukopenic mechanism in the underlying susceptibility of patients with mitochondrial disease or ataxia telangiectasia to infections.

## Methods

A detailed list of resources and reagents can be found in Supplemental Table 1.

### Animal models and husbandry.

Homozygous floxed *Sco1*, *Cox10*, and *Coa5* mice were used to generate hepatocyte-specific (*hep*) knockout models, as previously described (18). Briefly, floxed animals were crossed with mice in which *Cre* recombinase expression is driven by the albumin promoter (*Alb-Cre^tg/tg^*). *Cre*-positive, F1 progeny were then backcrossed to the appropriate homozygous floxed model to generate F2 litters, with roughly 25% of the resultant progeny exhibiting hepatocyte-specific loss of expression of the gene of interest.

Heart-specific *Ctr1-*knockout (*Ctr1^hrt^*) mice were generated according to Kim et al. (35). PCR genotyping of *Sco1* (18), *Cox10* (22), and *Ctr1* (35) mice was performed as previously described, and age-matched *flox/+* or *flox/flox* siblings served as Controls in this study. *Coa5* mice were genotyped as detailed below. Mice were housed under a 12-hour light/12-hour dark photoperiod in a temperature- and humidity-controlled facility and provided with food and water ad libitum.

### Generation of a conditional Coa5 mouse model.

Embryonic stem (ES) cells with floxed *Coa5* alleles were purchased from the Knockout Mouse Project (KOMP) Repository. Male chimera transmitter *Coa5* mice lacking the neomycin-resistance cassette and *lacZ* reporter gene were then generated fee-for-service at the Toronto Centre for Phenogenomics and shipped to the University of Saskatchewan. These males were crossed with C57BL/6N females, and the resultant *Coa5^loxP/wt^* progeny were intercrossed to yield homozygous *Coa5* mice with a residual FRT site and loxP sites that flank the second exon of the gene to allow for its deletion.

### Specimen collection.

Mice were weighed prior to being anesthetized with 2% isoflurane. Blood was then collected from the left ventricle of the heart using a 27-gauge needle, placed into a precoated K_2_EDTA tube, and inverted 10 times. After a 20-minute room temperature incubation, 100 μL of blood was retained on ice for further analyses while the remainder was used to isolate plasma by 2 sequential room temperature spins for 5 minutes at 1,000*g*.

Following blood collection, the thymus, heart, spleen, liver, kidney, and brain were harvested, and flash frozen on dry ice in preweighed Eppendorf tubes. Tubes were then weighed after collection, allowing for tissue wet weights to be calculated. Tissues were then powdered in a stainless steel mortar and pestle on dry ice and stored at –80°C for subsequent biochemistry, molecular biology, elemental, and genetic analyses.

### CBC count and peripheral blood smear.

Automated CBC analysis was performed on the fraction of retained blood using the COULTER Ac·T diff Analyzer (Beckman). All samples were run in duplicate. Alternatively, a spreader slide and 10 μL of blood were used to create a peripheral blood smear. Air-dried slides were then stained with Wright’s Giemsa using an immersion protocol. Briefly, slides were stained for 1 minute, rinsed for 5 minutes in phosphate buffer (pH 6.8) (made with potassium phosphate, monobasic 50.1% [w/w] and sodium phosphate, dibasic 49.9% [w/w]), washed briefly in running deionized water, dried, and coverslipped. The WBC count was determined by taking the average number of WBCs in 10 fields at ×40 high power and multiplying by 2.0 × 10^9^/L (67).

### Tissue perfusion, fixation, and histology.

Mice were anesthetized with 2%–3% isoflurane and oxygen, and perfused through the heart with 10 mL Hank’s balanced salt solution (HBSS, pH 7.2) containing 2.5% FBS at a flow rate of approximately 5 mL/min. Once the organs were cleared of blood, mice were perfused with another 10 mL of 10% formalin. The thymus, heart, spleen, and liver were harvested, stored overnight in 10% formalin, and then dehydrated and embedded in paraffin. Seven-micrometer cross sections were prepared, and slides were stained with H&E according to the manufacturer’s standard procedure. Sections were viewed and imaged using a scanning transmission light microscope (Leica).

### Adenovirus-related experiments.

HdAD encoding the *lacZ* transgene or vehicle (sterile Ringer’s solution) were administered via intracardiac (i.c.) or i.p. injection. We found that i.c. administration resulted in higher β-galactosidase expression in the liver, with less intense staining in other, peripheral tissues when compared with i.p. injection (Supplemental Figure 1B). We therefore used the i.c. route to administer 5.783 × 10^12^ *Sco1* HdAD particles/kg or an equivalent volume of vehicle to mice at 21–24 days of age. Mice were anesthetized with 2% isoflurane, the chest and abdomen were disinfected with 70% ethanol, and adenovirus or vehicle was administered with a 27-gauge needle by direct puncture of the left ventricle through the diaphragm following the drawback of fresh arterial blood. Blood and tissues were then collected when mice reached 47 days of age.

### Immunoblot analyses.

Powdered whole tissues were resuspended and homogenized on ice in extraction buffer (69) supplemented with a complete protease inhibitor cocktail (Roche) and 0.5 mM PMSF. Following a 30-minute incubation step on ice, lysates were clarified by centrifugation at 16,600*g* for 10 minutes at 4°C and equal amounts of protein (10–25 μg/lane) were electrophoresed in 4%–20% precast Tris-HCl gels (Bio-Rad). All proteins were then transferred onto nitrocellulose membranes under semidry conditions and blotted with the appropriate antibodies. Following incubation of membranes with secondary antibody, immunoreactive proteins were detected by luminol-enhanced chemiluminescence. For cultured cells, 2 × 10^5^ cells were seeded on a 6-well plate and treated with either DMSO or OXPHOS inhibitors, including rotenone (1 μM), malonate (1 mM), antimycin (1 μM), KCN (1 mM), and oligomycin (1 μM), for 24 hours. Cells were washed with PBS and harvested using a cell scraper. Cells were then lysed in RIPA buffer supplemented with protease inhibitor cocktail (Roche). After a 30-minute incubation in lysis buffer, cell lysates were collected by centrifugation at 14, 000*g* for 10 minutes at 4°C and equal amounts of protein (20 μg) were electrophoresed in 10% precast Bis-Tris gels (Life Technologies). Following semidry transfer of gels onto PVDF membranes, the membranes were blotted with indicated antibodies (anti-AFP made in-house and anti–β-actin from Sigma-Aldrich, A2228) and protein bands detected as above.

### Elemental analyses.

Samples were digested in 40% nitric acid by boiling for 1 hour in capped, acid-washed tubes, diluted in ultrapure, metal-free water, and analyzed by inductively coupled plasma optical emission spectroscopy (ICP-OES; Perkin Elmer, Optima 7300 DV) versus acid-washed blanks. Concentrations were determined from a standard curve constructed with serial dilutions of 2 commercially available mixed metal standards (Optima). Blanks of nitric acid with and without “metal-spikes” were analyzed to ensure reproducibility.

### qPCR analyses.

Total RNA was extracted with TRIzol Reagent (Thermo Fisher Scientific, 15596026) and quantified using a DS-11 Series spectrophotometer (Denovix Inc). cDNA was synthesized using Superscript IV Reverse Transcriptase and Oligo(dT)20 primers from Thermo Fisher Scientific. qPCR was performed using SsoFast EvaGreen Supermix (Bio-Rad) on a CFX384 Touch Real-Time PCR Detection System (Bio-Rad). Data were analyzed according to the delta delta Ct method and *Afp* levels were normalized against *Gadph* abundance.

### ATP quantitation.

ATP levels in *Sco1^hep^*, *Cox10^hep^*, *Coa5^hep^*, and age-matched littermate Control livers were measured using a luminescence detection kit, as per the manufacturer’s instructions. Briefly, 10 mg of powdered liver tissue was homogenized in 2.5% trichloroacetic acid, sonicated on ice, and neutralized with Tris-acetate buffer. Signal intensities were read using a Luminex multimode plate reader. Arbitrary luminescence units in *hep* livers are represented as the fold change relative to the appropriate Control group.

### Tail vein injections.

Plasma for tail vein injections was aseptically pooled, aliquoted, and stored at –80°C prior to the beginning of an experiment. Control mice were injected via the tail vein between 24 and 27 days of age with a 27-gauge needle, after tails had been warmed for 5–10 minutes under a heat lamp and subsequently cleaned with 70% ethanol to prevent infection. Animals were injected with the maximum recommended volume of 10 mL/kg or 100–200 μL of plasma isolated from Control or *Sco1^hep^* mice, or with 1 μg of rAFP or albumin, a closely related family member. Mice were injected every 72 hours thereafter for a 28-day period. Blood and tissues were collected 3 days after the eighth injection.

### PBMC isolation and culture.

Mouse blood was collected as described above. Human blood was obtained from 3 consenting, healthy volunteers (30–50 years of age) and used in experiments approved by the Research Ethics Board at the University of Saskatchewan. Both mouse and human PBMCs were isolated by Ficoll Plaque Plus density centrifugation, and cultured at a concentration of 1 × 10^6^ cells/mL in RPMI 1640 media supplemented with 20% FBS and 100 IU IL-2. All related experiments were carried out 1 to 3 days after PBMCs were initially isolated.

PBMC viability assays were quantified by counting the number of live and dead/dying cells in bright-field photomicrographs acquired at ×60 magnification. Cell counting was performed by an observer who was blind to the treatment conditions, using the Point Tool in ImageJ (https://imagej.nih.gov/ij/). Cells were defined as alive or dead/dying based on their size and morphology. Round cells with a diameter of 5 μm or smaller and with an intact cell membrane were defined as live cells. Round cells with a diameter of 3 μm or smaller, and cells with a diameter of 3 μm or larger that exhibit obvious signs of cell blebbing (a hallmark of apoptosis), were defined as dead/dying cells. For each photomicrograph, the percentage of dead cells was calculated as (number of dead or dying cells)/(number of dead or dying cells + number of live cells) × 100. Three to 13 images were quantified per treatment condition, acquired from 5 independent experiments.

### Plasma fractionation and PBMC treatment.

Plasma pools were generated for *Sco1^hep^*, *Cox10^hep^*, and age-matched littermate Control animals. Plasma from mice fed an HF diet (28) was also obtained, pooled, and used as an additional control. PBMCs were cultured as described above in media containing 20% FBS or 17.5% FBS and 2.5% Control, *hep*, or HF plasma.

To determine whether the bioactive factor in *hep* plasma was a protein and the nature of that protein, plasma from Control and *Sco1^hep^* mice was first boiled for 5 minutes at 98°C or treated with trypsin for 1 hour. PBMCs were then cultured as described above in media containing 17.5% FBS plus 2.5% untreated, boiled, or trypsinized plasma from Control or *Sco1^hep^* mice. Control and *hep* plasma was then separated by size using centrifugal 50 kDa filters (Amicon) and by glycan content using the Qproteome Total Glycoprotein Kit, with both steps being done according to the manufacturer’s instructions. PBMCs were cultured as described above in media supplemented with 17.5% FBS containing 2.5% of each plasma fraction.

For AFP depletion experiments, a volume of plasma equivalent to 2.5% v/v from Control or *Sco1^hep^* mice was incubated with 1 μg mouse anti-AFP and rotated at 4°C overnight. Protein A Dynabeads (250 μL; Invitrogen, 10002D) were then added to the plasma and incubated at room temperature for 2 hours. Mouse anti-SLC25A3 was used as an IgG isotype control. Antibody-bead complexes were collected on a magnet, and the resultant plasma was used to treat PBMCs as described above. Control and *Sco1^hep^* plasma that had been incubated with an equivalent volume of PBS served as internal controls.

### MS and differential analysis.

Quantitative MS analyses were conducted fee-for-service by the Proteomics platform at the McGill University Health Centre. Briefly, 2 μg of the >50 kDa glycosylated plasma fraction from Control, *Sco1^hep^*, *Cox10^hep^*, and HF mice was trypsinized and analyzed using a Thermo Fisher Scientific Ultimate 3000 HPLC and Orbitrap Fusion MS: Quadrupole-Orbitrap-Linear ion trap hybrid. Raw data were then mined with Pinnacle (http://www.optystech.com/index.html#) and the spectral counts for each peptide were quantified (Supplemental Table 2). Only proteins for which 2 unique peptides were detected were included in the final analyses, and quantitative data for each experimental group represent the average of 2 independent MS runs.

Liquid chromatography–MS (LC-MS) differential analysis was performed using R (70). The package DESEQ2 (71) was used to test for differential expression, the package data.table (72) was used for data manipulation, and ggplot2 was used to generate plots (73). Raw data were imported (20190508_LCMS_raw_data_sheet1; Supplemental Table 2) and counts were converted to integers. Counts with NA were converted to zero. One count was added to all samples. The count matrix was prepared (20190508_input_sheet2; Supplemental Table 2) and DESeq2 was performed on *hep* versus Control. A false discovery rate (FDR) of 0.1 was applied to report significant (upregulated/downregulated) versus nonsignificant (NS) hits (20190508_results_sheet3; Supplemental Table 2).

### Flow cytometry.

PBMCs isolated from Control and *Sco1^hep^* blood were stained with an anti-CD45, -CD3, -CD4, -CD25, -CD44, and -CD69 antibody cocktail or with an anti-CD3 and -CD4 antibody cocktail that included 7AAD (Thermo Fisher Scientific, 006993-50) and annexin V (BD Biosciences) staining solutions. Briefly, PBMCs were resuspended in PBS with 2.5% FBS at 1 × 10^6^ cells/mL. Cells were blocked with anti–mouse CD16/CD32 Fc block for 15 minutes and then incubated with the primary antibody cocktail for 30 minutes. For staining with annexin V, PBMCs were resuspended and blocked as described above, and then incubated with anti-CD3 and anti-CD4 for 20 minutes. 7AAD was subsequently added to the staining mixture and further incubated for 10 minutes. Cells were then centrifuged for 5 minutes at 1,000*g*, resuspended in annexin V binding buffer, and stained according to the manufacturer’s instructions. Following either staining procedure, cells were fixed in a solution of 1% formalin with RBC lysing solution for 15 minutes. All events were analyzed using a CellFlex Flow Cytometer (Beckman Coulter). Dead cells were excluded based on forward and side light scattering. All antibody staining procedures took place at room temperature in the dark.

### Statistics.

All statistical analyses were performed using GraphPad Prism 8.0 or 9.0. Data are reported as mean ± SEM. For selection of appropriate statistical tests, data were assessed by histogram or Tukey plot to detect a normal Gaussian distribution. After verifying a Gaussian distribution, statistical differences between 2 groups were assessed using a 2-tailed Student’s *t* test and between 3 or more groups with 1-way ANOVA and Tukey’s or Sidak’s post hoc test. For the qPCR analyses in Supplemental Figure 5, differences in *Afp* mRNA abundance between the Control and *Ctr1^hrt^* hearts were assessed using a nonparametric Mann-Whitney *U* test, as the data were not normally distributed. For data with 2 independent variables, 2-way ANOVA and Bonferroni’s post hoc test was used. *P* values of less than 0.05 were considered significant. Statistical parameters can be found in the figure legends.

### Study approval.

All experiments on F2 *hep* animal models were approved by the Animal Ethics Review Board at the University of Saskatchewan (AUP no. 20100091), while those involving the *Ctr1* mouse model were conducted in accordance with NIH *Guide for the Care and Use of Laboratory Animals* (National Academies Press, 2011) and approved by the Institutional Animal Care and Use Committee at the University of Maryland, College Park (AUP no. R-APR-18-14).

## Author contributions

SCL and KAJ conceived the original study, while KAJ, PAC, BEK, HYMC, VMG, and SCL contributed to its further refinement. Data were collected by KAJ, ZNB, AB, MAH, OY, PAC, SG, KB, SY, AH, and CL, and analyzed by KAJ, SG, KB, CL, OHMHA, and SY. JD, PN, KM, CS, and GNI provided guidance, reagents, or samples invaluable to experimental advancement. The manuscript was written by SCL, BJB, and KAJ and edited by PAC, BJB, BEK, HYMC, and VMG.

## Supplementary Material

Supplemental data

Supplemental table 1

Supplemental table 2

## Version 1 10/27/2022

In-Press Preview

## Version 2 01/03/2023

Electronic publication
