# Supplementary material for: Mitochondrial dysfunction reactivates α-fetoprotein expression that drives copper-dependent immunosuppression in mitochondrial disease models
Source: J Clin Invest. 2023 Jan 3;133(1):e154684. doi: 10.1172/JCI154684 (PMC9797342; doi:10.1172/JCI154684)
Supplement: Supplemental data [file jci-133-154684-s007.pdf]

Mitochondrial dysfunction reactivates α-fetoprotein expression that drives copper-dependent immunosuppression in mitochondrial disease models

Kimberly A. Jett[1], Zakery N. Baker[1], Amzad Hossain[1], Aren Boulet[1], Paul A. Cobine[2], Sagnika Ghosh[3], Philip Ng[4], Orhan Yilmaz[1], Kris Barreto[5], John DeCoteau[5], Karen Mochoruk[5], George N. Ioannou[6,7,8], Christopher Savard[6,7,8], Sai Yuan[9], Osama H.M.H. Abdalla[10,11], Christopher Lowden[10,11], Byung-Eun Kim[9], Hai-Ying Mary Cheng[10,11], Brendan J. Battersby[12], Vishal M. Gohil[3] and Scot C. Leary[1,13,14,*]

Supplemental Figures

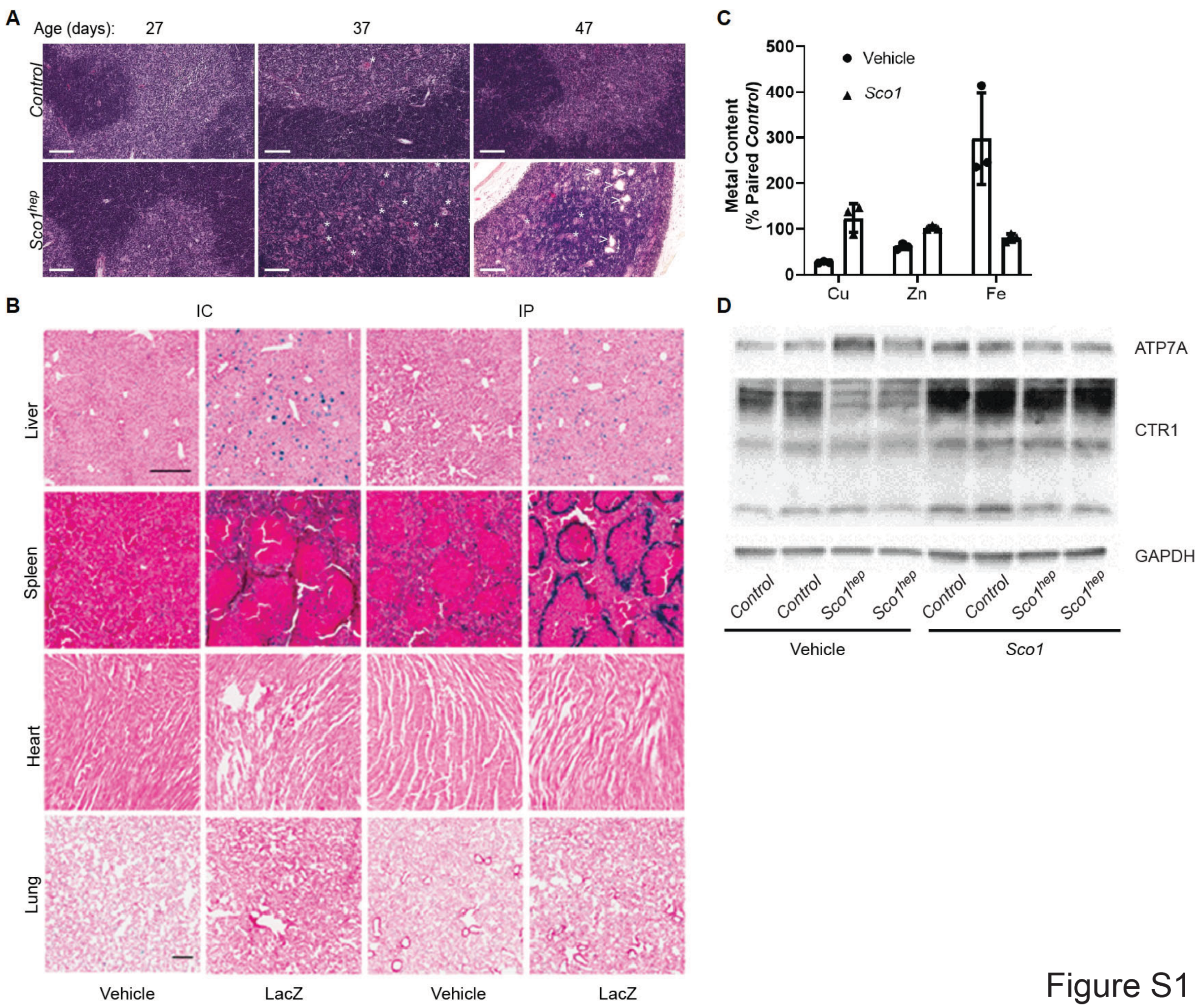

Figure S1. *A)* The *Sco1^{hep}* thymus exhibits progressive thinning of the cortex from P27 to P47, with disruption of the cortico-medullary boundary, accumulation of tingible body macrophages (denoted with a *) and increased vascularity (denoted with a >). Scale bar, 100µm. *B)* LacZ staining in the liver (4X), spleen (4X), heart (4X) and lung (2X) upon intracardiac (I*C*) or intraperitoneal (IP) administration of vehicle or helper-dependent adenovirus. *C&D)* Restoration of *Sco1* expression in the *Sco1^{hep}* liver normalizes *C)* metal ion levels (t- test, n=3, Cu and Fe, $p< 0.01$; Zn, $p< 0.05$) and *D)* CTR1 abundance. *Control* refers to wild-type littermates.

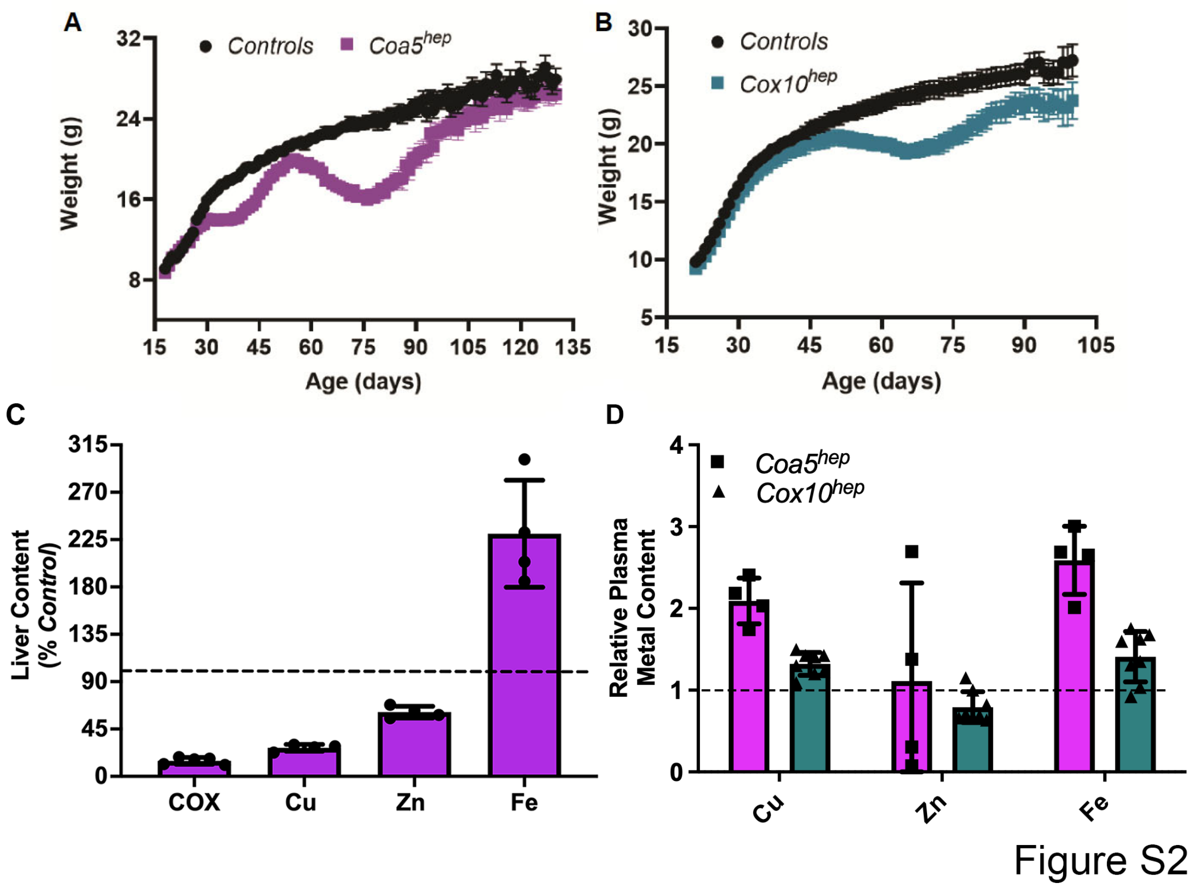

Figure S2. Change in body weight (g*)* over time in *A) Coa5*^{hep} (*Control*, n=23-64; *Coa5*^{hep}, n=6-31) and *B) Cox10*^{hep} (*Control*, n=11-31; *Cox10*^{hep}, n=5-16) mice. *C) Coa5*^{hep} livers have a severe COX (t-test, n=5) and copper deficiency relative to livers from *Control* littermates. *D)* Plasma copper, iron and zinc levels in *Coa5*^{hep} (t-test, n=4) and *Cox10*^{hep} (t-test, n=8) plasma relative to age-matched, littermate *Controls*.

Figure S3

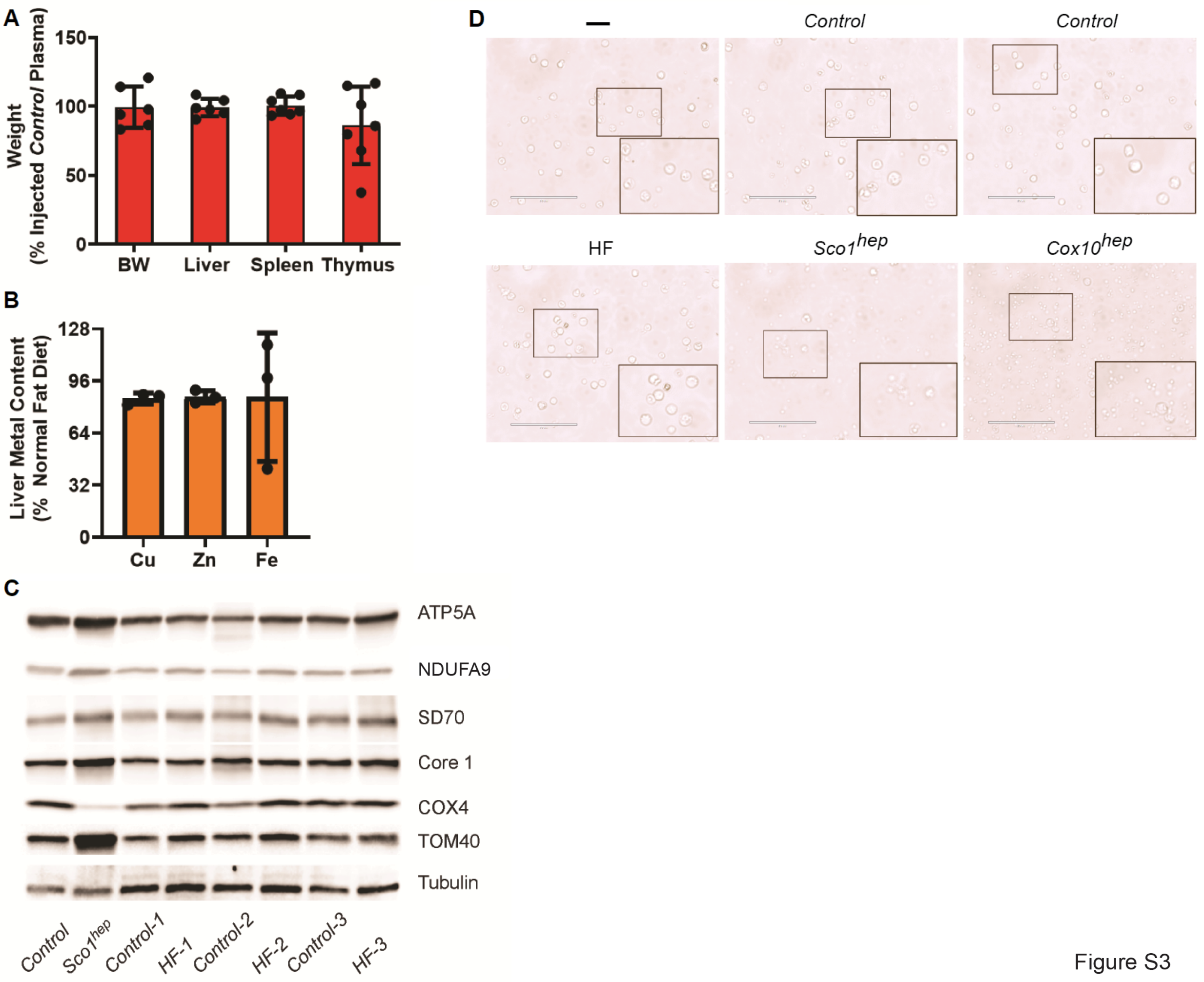

Figure S3. *A)* Body and organ weight are unaffected in *Control* mice injected with *Sco1*^{hep} plasma relative to those injected with *Control* plasma (t-test, n=6-7). *B)* Metal content (t-test, n=3) and *C)* OXPHOS subunit abundance are unaltered in livers from mice fed a high fat (HF) diet compared to those fed normal chow. *Control* and *Sco1*^{hep} liver extracts were included for comparative purposes and tubulin served as an internal loading control. *D)* Lower magnification showing a greater number of PBMCs, with black boxes depicting the region of interest shown in Figure 3B.

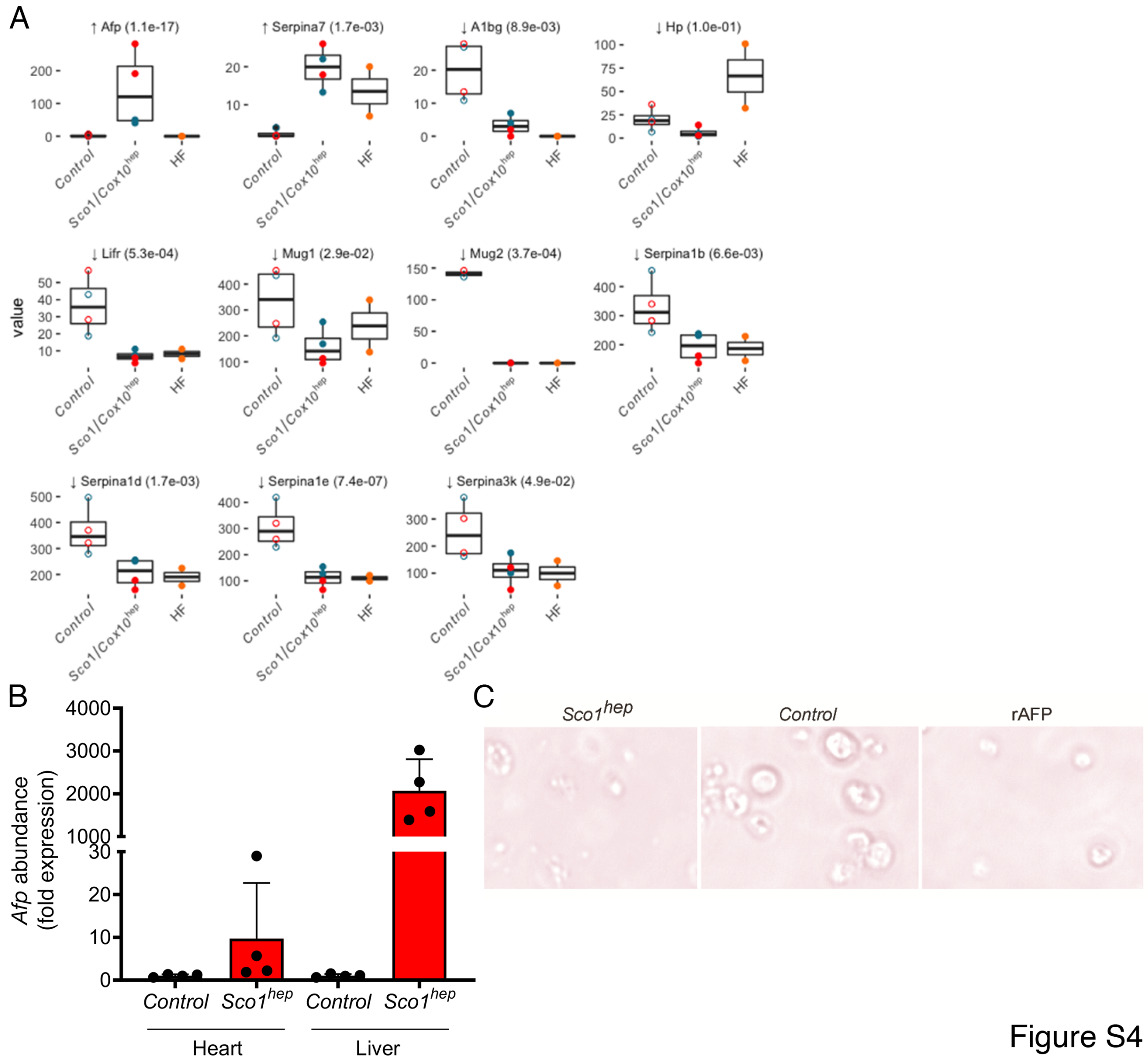

Figure S4

Figure S4. *A)* Individual box plots of significantly up- or downregulated plasma proteins in *hep* compared to *Control* mice. Red and blue circles denote data from the *Sco1* and *Cox10* models, respectively (open circles, *Control* animals; closed circles, *hep* animals). HF denotes plasma from mice fed a high fat diet. *B) Afp* mRNA levels are significantly higher in the *Sco1*^{hep} liver (ANOVA; $p < 0.02$) but not the heart, when compared to *Control* tissues from age-matched littermates (n=4, all tissues and genotypes). Transcript levels were normalized to *Gapdh* mRNA abundance. *C)* PBMC viability is similarly reduced upon treatment with *Sco1*^{hep} plasma or recombinant AFP (rAFP, 1μg).

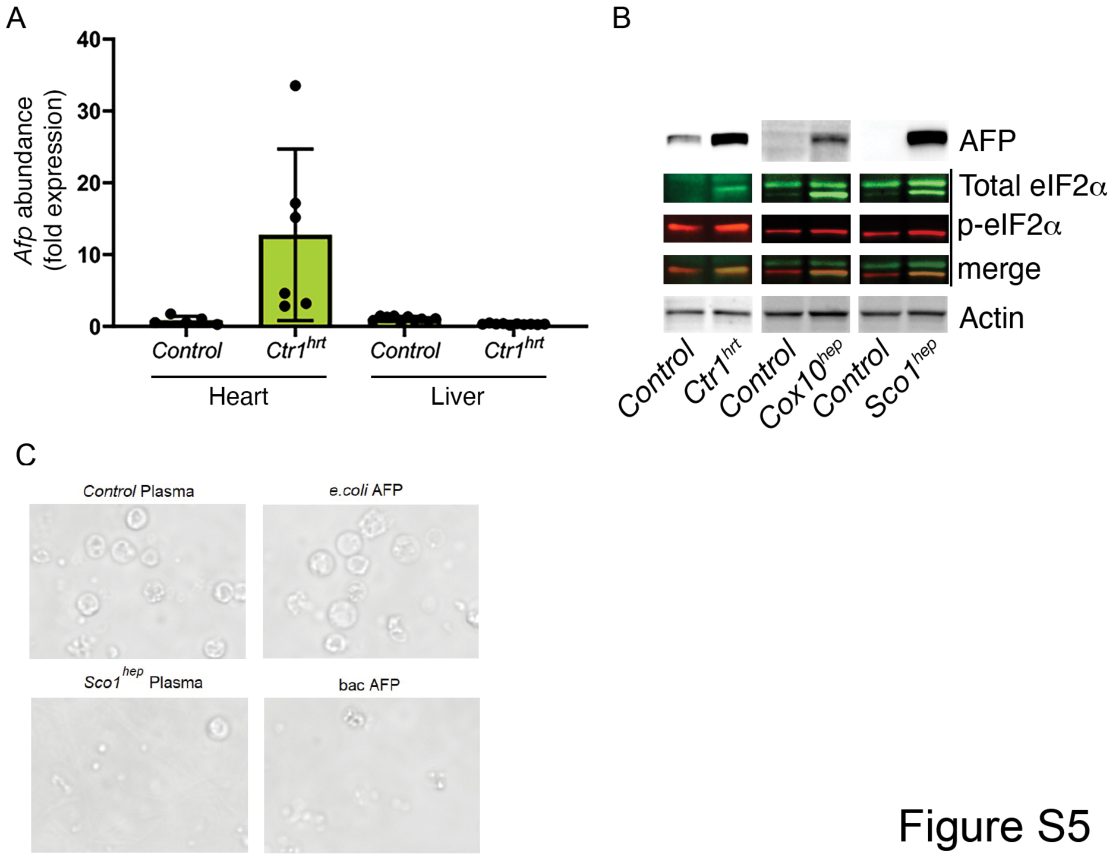

Figure S5. *A) Afp* mRNA levels are significantly higher in the *Ctr1[hrt]* heart (ANOVA; $p < 0.005$) but not the liver, when compared to *Control* tissues from age-matched littermates (heart, n=6; liver, n=10 for both genotypes). Transcript levels were normalized to *Gapdh* mRNA abundance. *B)* The *Ctr1[hrt]* heart has elevated levels of AFP and the ISR marker phospho-eIF2α. Equal amounts of *Control* and *hep* liver extracts from the *Sco1* and *Cox10* lines were included in these analyses for comparative purposes. N.B. original data for actin, total and phospho-eIF2α livers from both hep models are shown in Figure 2E. *C)* The viability of PBMCs isolated from *Control* mice is reduced when co-cultured with AFP produced by baculovirus (bac AFP, the rAFP used in Figure S4C of this study) but not with AFP isolated from *E. coli*. *Control* and *Sco1[hep]* plasma were included in these analyses as negative and positive controls, respectively.

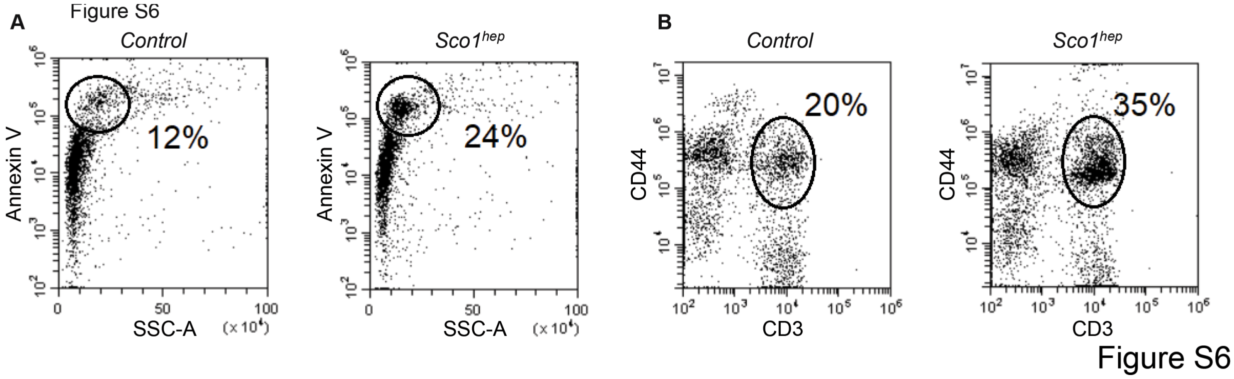

Figure S6. Representative flow plots of peripheral PBMCs show that *Sco1*^{hep} mice show have a higher percentage of cells positive for the cell surface expression of *A)* the apoptotic marker Annexin V and *B)* the activation marker CD44.
