# Supplementary material for: Mitochondrial dysfunction reactivates α-fetoprotein expression that drives copper-dependent immunosuppression in mitochondrial disease models
Source: J Clin Invest. 2023 Jan 3;133(1):e154684. doi: 10.1172/JCI154684 (PMC9797342; doi:10.1172/JCI154684)
Supplement: Supplemental table 1 [file jci-133-154684-s008.pdf]

Supplemental Table 1. Description, details and origin of key resources used in this study.

Reagent or Resource	Source	Identifier
**Antibodies**		
SCO1	Rabbit polyclonal	In-house
GAPDH	Cell Signaling	2118
ATP7A	In-house	(18)
CTR1	GenScript	In-house
eIF2α	Cell Signaling	9722
phospho-eIF2α	Cell Signaling	9721
Actin hFAB rhodamine	Cell Signaling	12004163
SD70	Mitoscience	MS204
Core 1	Mitoscience	MS303
ATP5A	Abcam	Ab14748
NDUFA9	Mitoscience	MS111
COX1	Mitoscience	MS404
COX4	Mitoscience	MS407
TOM40	Proteintech	18409-1-AP
AFP	Abcam	Ab46799
Tubulin	Santa Cruz	sc-5286
CD16/CD32 (Fc Block)	BD Biosciences	553141
CD3	BD Biosciences	565643
CD4	BD Biosciences	557308
CD8	BD Biosciences	553030

CD25	BD Biosciences	552880
CD44	BD Biosciences	563736
CD69	BD Biosciences	561240
FITC Annexin V	BD Biosciences	556419
Annexin V Binding Buffer, 10X	BD Biosciences	556454
**Bacterial and Virus Strains**		
XL1-Blue Competent Cells	Agilent	200249
HDΔ28E4LacZ	In-house	(20, 21)
HDΔ24.7E4-pepck-mSCO1	In-house	(20, 21)
**Chemicals, Peptides, and Recombinant Proteins**		
Ammonium persulfate	BioShop	AMP001.100
Acrylamide	Sigma	A8887-500G
Bathocuproinedisulfonic acid disodium salt	Sigma	B1125-1G
Calcium chloride dihydrate	EM Science	B10070-34
Cupric chloride dihydrate	BioShop	CUC002.100
Cupric sulfate pentahydrate	Sigma	C-8027
Dimethyl sulfoxide	BioShop	DMS666.50
Ethylenediamine	BioShop	EDT001.500
Ethylenediamine tetraacetic acid dipotassium salt dihydrate	Fisher	BP19-500
Glycine	BioShop	GLN002.5
L-Histidine	Sigma	H9288

Maraviroc	Sigma	PZ0002
PMSF	BioShop	PMS123.5
Potassium chloride	EM Science	PX1405-1
SDS	BioShop	SDS001.1
Sodium chloride	BioShop	SOD002.5
Potassium phosphate, monobasic	BioShop	PPM302.500
Sodium phosphate, dibasic	BioShop	SPD579.1
Triton-X 100	BioShop	TRX777.5
Tween-20	Fisher	BP337-500
Trypan blue solution (0.4%)	Sigma	T8154
Tris	BioShop	TRS001.5
Skim Milk (Blotto)	US Biological	S1013-90A
TEMED	BioShop	TEM001.5
Complete Protease Inhibitor Cocktail (PIC)	Roche	11 836 145 001
Hydrogen peroxide solution	Sigma	216763
Albumin (Bovine)	BioShop	ALB001.500
Dithiotreitol (DTT)	Fisher	BP172-5
Oxaloacetic acid (OAA)	Sigma	O4126-1G
Proteinase K from *Tritirschium album*	Sigma	P2308-100MG
Cytochrome *c*	Sigma	C-7752
Agarose	BioShop	AGA002.1

SYBR Safe DNA gel stain	Invitrogen	S33102
Generuler (100bp)	Thermo	SM0241
p-Coumaric acid	Sigma	C9008-5G
Luminol	Sigma	A8511-5G
Ponceau S	Fisher	40058003755
Recombinant Mouse α-fetoprotein (AFP)	Mybiosource	MBS717717
Trichloroacetic acid (TCA)	Sigma	T0699
RPMI 1640 Medium	GE	SH30096.01
10X PBS	Fisher	BP3994
FBS	Gibco	12483020
Recombinant human interleukin-2 (IL-2)	Stem Cell	78036.3
Ficoll Paque Plus	Sigma	17-1440-02
Hank's Balanced Salt Solution (HBSS) (10X)	Gibco	14065056
Isoflurane	Fresenius Kabi	CP0406V2
Harris Hematoxylin	Leica	3801562
Eosin Y	Leica	3801602
**Critical Commercial Assays**		
Phire Animal Tissue Direct PCR kit	Thermo	#F-140WH
Mouse AFP Quantikine ELISA Kit	R&D Systems	MAFP00
ENLITEN® ATP Assay System Bioluminescence Detection Kit for ATP Measurement	Promega	FF2000
Qproteome Total Glycoprotein Kit	Qiagen	37541

**Experimental Models:**<br><br>*<u>Mouse strains</u>*		
Alb-cre	Jax	#003574
Sco1	In-house	(18)
Coa5	KOMP - ES cells; Toronto Centre for Phenogenomics - mice	
Cox10	Jax	#024697
Ctr1	In-house	(35)
<u>Cell lines</u>		
MCH58 human skin fibroblasts	Dr. Eric Shoubridge, McGill University	
C2C12 murine myoblasts	Dr. Miriam L. Greenberg, Wayne State University	
Control B-lymphocytes	Coriell Institute	ND11500
**Oligonucleotides**		
Sco1-F<br>ATGGAATCCCTTCCTTGCTTC	In-house	(18)
Sco1-R1<br>TCAACCTCAACATTTCACGACGGTATT	In-house	(18)
Sco1-R2	In-house	(18)

ACCTAAAAGTGGGGCTTCCTGAAAACTAA		
Cox10-F<br><br>GAGAGGAGTCAAGGGGACCT	This study	N/A
Cox10-R1<br><br>GGCCTGCAGCTCAAAGTGTA	This study	N/A
Cox10-R2<br><br>CAAAGAGGGCTCACTTCTTGC	This study	N/A
Coa5-F<br><br>GAGCTCTCATGCACAGCAAG	This study	N/A
Coa5-R1<br><br>TTCAAGTCGTGGAATGGTAGC	This study	N/A
Coa5-R2<br><br>GCTGCTAGGACCAAATCCTG	This study	N/A
Ctr1-P1<br><br>AATGTCCTGGTGCGTCTGAAA	In-house	(35)
Ctr1-P2<br><br>GCAGTAGATAAAAGCCAAGGC-30	In-house	(35)
Ctr1-P3<br><br>AAAAACCACTATTCAGAGACTG	In-house	(35)
Afp-F<br><br>AGTTGCAAAGCACATGAAGA	IDT	
Afp-R<br><br>AAGCACTCCTCCTTGTTGTC	IDT	
Gapdh-F	IDT	

CATGGCCTTCCGTGTTCCTA		
Gapdh-R<br><br>CCTGCTTCACCACCTTCTTGA	IDT	
ATF6-F<br><br>TCGCCTTTTAGTCCGGTTCTT	IDT	
ATF6-R<br><br>GGCTCCATAGGTCTGACTCC	IDT	
**Software & Algorithms**		
GraphPad Prism 8.0/9.0		
CytExpert	Beckman-Coulter	
ImageJ		
